# The chemerin receptor 23 agonist, chemerin, attenuates monosynaptic C-fibre input to lamina I neurokinin 1 receptor expressing rat spinal cord neurons in inflammatory pain

**DOI:** 10.1186/1744-8069-10-24

**Published:** 2014-04-09

**Authors:** Allen C Dickie, Carole Torsney

**Affiliations:** 1 Centre for Integrative Physiology, The University of Edinburgh, Edinburgh, UK

**Keywords:** Dorsal horn, Lamina I, NK1R, Projection neurons, Chemerin, ChemR23, Inflammatory pain, Resolvins

## Abstract

**Background:**

Recent evidence has shown that the chemerin receptor 23 (ChemR23) represents a novel inflammatory pain target, whereby the ChemR23 agonists, resolvin E1 and chemerin, can inhibit inflammatory pain hypersensitivity, by a mechanism that involves normalisation of potentiated spinal cord responses. This study has examined the ability of the ChemR23 agonist, chemerin, to modulate synaptic input to lamina I neurokinin 1 receptor expressing (NK1R+) dorsal horn neurons, which are known to be crucial for the manifestation of inflammatory pain.

**Results:**

Whole-cell patch-clamp recordings from pre-identified lamina I NK1R+ neurons, in rat spinal cord slices, revealed that chemerin significantly attenuates capsaicin potentiation of miniature excitatory postsynaptic current (mEPSC) frequency, but is without effect in non-potentiated conditions. In tissue isolated from complete Freund’s adjuvant (CFA) treated rats, chemerin significantly reduced the peak amplitude of monosynaptic C-fibre evoked excitatory postsynaptic currents (eEPSCs) in a subset of lamina I NK1R+ neurons, termed chemerin responders. However, chemerin did not alter the peak amplitude of monosynaptic C-fibre eEPSCs in control tissue. Furthermore, paired-pulse recordings in CFA tissue demonstrated that chemerin significantly reduced paired-pulse depression in the subset of neurons classified as chemerin responders, but was without effect in non-responders, indicating that chemerin acts presynaptically to attenuate monosynaptic C-fibre input to a subset of lamina I NK1R+ neurons.

**Conclusions:**

These results suggest that the reported ability of ChemR23 agonists to attenuate inflammatory pain hypersensitivity may in part be due to a presynaptic inhibition of monosynaptic C-fibre input to lamina I NK1R+ neurons and provides further evidence that ChemR23 represents a promising inflammatory pain target.

## Background

Inflammatory pain is a common clinical problem, however many currently used treatments, such as opioids or cyclooxygenase (COX) inhibitors, lack efficacy and/or exhibit undesirable side effects [1,2]. Therefore, the development of new analgesics that are both efficacious and lack side effects is a key challenge for pain research.

Lamina I neurokinin 1 receptor expressing (NK1R+) neurons, a significant proportion of which are projection neurons [3-6], are known to be crucial for the manifestation of inflammatory pain hypersensitivity. Selective ablation of these neurons by spinal administration of a substance P–saporin conjugate significantly attenuates hyperalgesia and allodynia in inflammatory pain models [7]. C-fibre nociceptors also play an essential role in the development of inflammatory pain. Transgenic mice in which Na_*v*_1.8 expressing nociceptors, which includes the majority of C-fibres, have been selectively eliminated, fail to develop inflammatory pain hypersensitivity [8]. Lamina I NK1R+ neurons predominantly receive monosynaptic C-fibre input [9-11] and some of this input may be potentiated in inflammatory pain [12]. Therefore, exploring potential strategies to modulate C-fibre input to these neurons could offer insights into novel inflammatory pain treatments.

Recent evidence has suggested that the chemerin receptor 23 (ChemR23), a G_*α*_i associated G-protein coupled receptor (GPCR), may be a promising target for the development of novel inflammatory pain treatments. The lipid mediator, resolvin E1 (RvE1), which acts via ChemR23 [13], has been shown to reduce thermal and mechanical hypersensitivity in the complete Freund’s adjuvant (CFA) and carrageenan inflammatory pain models [14]. Furthermore, RvE1 attenuates the second phase of the formalin response, in a manner that is comparable to morphine and NS-398, a commonly used COX-2 inhibitor, but at a substantially reduced dose [14]. Chemerin, a natural ChemR23 ligand [15,16], has also been shown to inhibit the second phase of the formalin test [14]. Interestingly, basal mechanical and thermal thresholds and the first phase of the formalin test are unaltered by RvE1 administration, suggesting that strategies which target ChemR23 may promisingly attenuate maladaptive/chronic pain without altering acute protective pain.

ChemR23 agonists have been found to have both peripheral and central actions. In the periphery, RvE1 reduces carrageenan-induced oedema, neutrophil infiltration and proinflammatory cytokine and chemokine expression [14]. Electrophysiological study of central actions, in spinal cord slices, have established that RvE1 and chemerin can ‘normalise’ potentiated spinal cord responses, without altering basal synaptic transmission in the dorsal horn. Specifically, application of chemerin or RvE1 abolishes capsaicin potentiation of spontaneous excitatory postsynaptic current (sEPSC) frequency, while RvE1 has also been shown to inhibit TNF- *α* mediated potentiation of sEPSC frequency and NMDA currents, in unidentified lamina II neurons [14]. However, as these findings were obtained using sEPSC recordings from unidentified neurons, it is not known where in the spinal cord network or upon which neuronal subtype these central effects are mediated.

Anatomical studies in mice demonstrate that ChemR23 is expressed both peripherally, in dorsal root ganglia (DRG) and centrally, on the central terminals of primary afferent fibres and also on spinal cord neurons [14]. Microarray studies, performed using rat tissue, also report ChemR23 mRNA expression in DRG neurons and in the dorsal horn, with expression levels being unaltered in CFA inflammation [17]. In mice, almost one third of DRG neurons express ChemR23 and there is a large degree of overlap between ChemR23 and transient receptor potential subtype vanilloid 1 (TRPV1) channel expression, with ∼45% of ChemR23 expressing neurons also expressing TRPV1 and ∼60% of TRPV1 expressing (TRPV1+) neurons also expressing ChemR23 [14]. In lamina I of the dorsal horn, ChemR23 is expressed on the central terminals of substance P containing (substance P+) afferents [14]. Lamina I NK1R+ neurons are known to be targeted by both TRPV1+ [18-20] and substance P+ afferents [18,21], with many C-fibres, which are the predominant type of input received by these neurons [9-11], expressing TRPV1 and/or substance P [22-28]. Therefore, given the co-expression of ChemR23 with TRPV1 and substance P, it is likely that some of the monosynaptic C-fibre input to lamina I NK1R+ neurons will also express ChemR23. As such, it is possible that the ability of ChemR23 agonists to attenuate inflammatory pain hypersensitivity could in part be due to an inhibition of C-fibre input to these key spinal cord output neurons.

In this study we aimed to determine whether the ChemR23 agonist, chemerin, could attenuate capsaicin potentiation of miniature excitatory postsynaptic current (mEPSC) frequency in lamina I NK1R+ neurons. Furthermore, we have investigated whether chemerin can modulate monosynaptic C-fibre input to these neurons during inflammatory pain. While much of the interest in targeting ChemR23 has revolved around the use of RvE1, it should be noted that RvE1 is not commercially available, therefore it was not possible to investigate the effect of this compound upon the synaptic input to lamina I NK1R+ neurons.

## Results

### Chemerin attenuates capsaicin potentiation of mEPSC frequency in lamina I NK1R+ neurons, but is without effect in non-potentiated conditions

**Figure 1:**
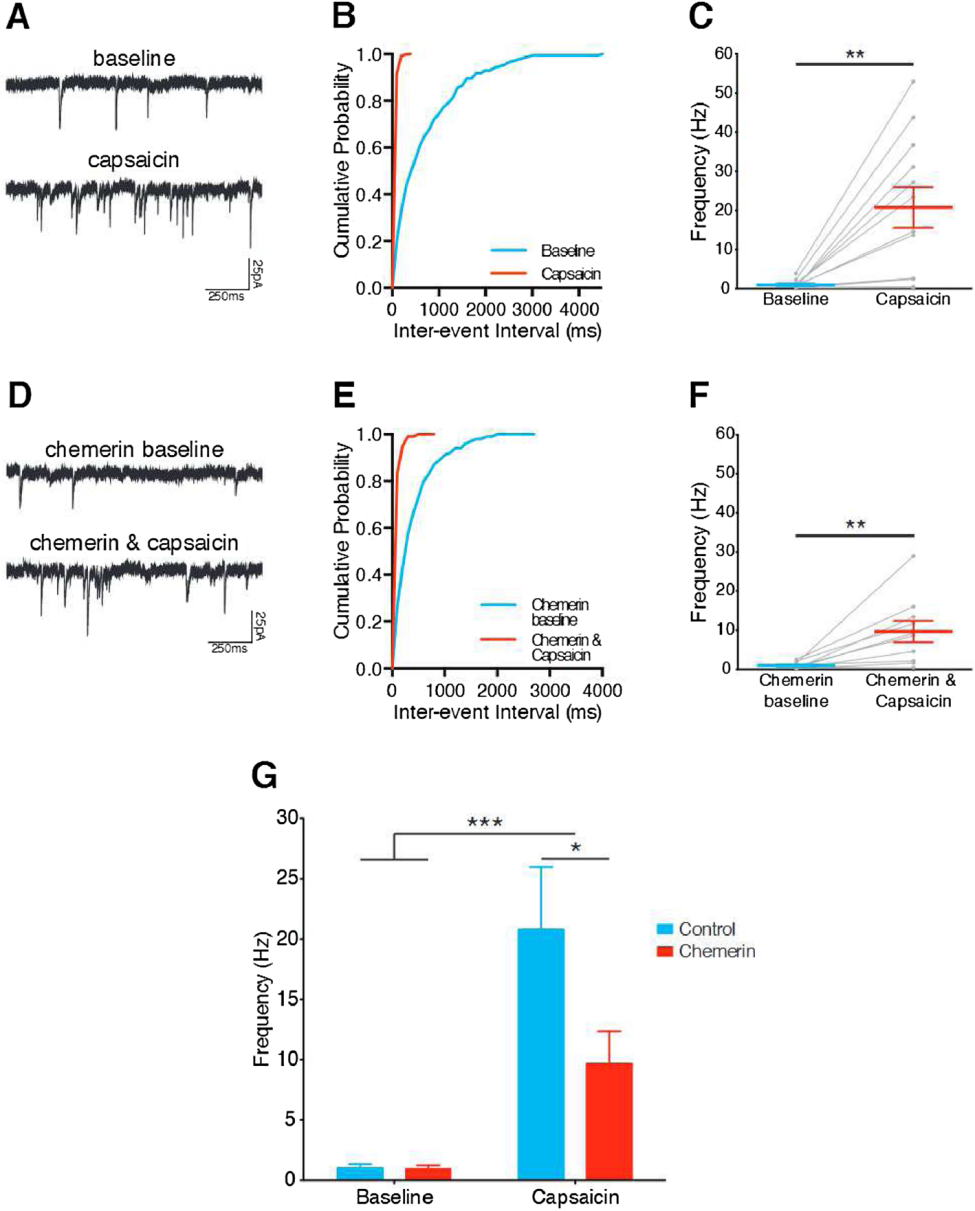
**Chemerin attenuates capsaicin potentiation of mEPSC frequency in lamina I NK1R+ neurons.** **A** and **D**. Representative mEPSC traces recorded before (top) and during (bottom) capsaicin (1 *μ*M) application in the absence **(A)** and presence **(D)** of chemerin (100 ng/ml). **B** and **E**. Example cumulative probability plots demonstrate the significant leftward shift in mEPSC inter-event intervals that results from capsaicin applied alone **(B)** or in the presence of chemerin **(E)** (both P < 0.00001, Kolmogorov-Smirnov 2-sample test). **C** and **F**. Application of capsaicin significantly increases mEPSC frequency when applied alone (**C**, P = 0.002, paired t-test, n = 12) and during chemerin application (**F**, P = 0.008, paired t-test, n = 10). **G**. Capsaicin significantly increases mEPSC frequency in the presence and absence of chemerin (P = 0.0001, 2-way repeated measures ANOVA), however this increase is significantly attenuated by chemerin (P = 0.031, Bonferroni post-tests). All data presented as mean ± SEM, grey points and lines in **C** and **F** indicate trajectories for individual neurons. ∗ P < 0.05, ∗∗ P < 0.01, ∗∗∗ P < 0.001.

Whole-cell patch-clamp electrophysiology was used to record mEPSCs from lamina I NK1R+ neurons in spinal cord slices from control rats, before and during capsaicin application, in either the presence or the absence of the ChemR23 agonist, chemerin (example traces in Figure 1A and D). Bath application of 1 *μ*M capsaicin, without chemerin, for 5 mins resulted in a significant leftward shift in the distribution of mEPSC inter-event intervals, indicating increased frequency, in 10/12 neurons, while in 2/12 there was no change (Kolmogorov-Smirnov 2-sample test, example shown in Figure 1B). Overall, capsaicin alone significantly increased mEPSC frequency in lamina I NK1R+ neurons (P = 0.002, paired t-test, n = 12, Figure 1C). When capsaicin was applied in the presence of chemerin (100 ng/ml), there was a significant leftward shift in the distribution of mEPSC inter-event intervals in 9/10 neurons and no change in 1/10 (Kolmogorov-Smirnov 2-sample test, example shown in Figure 1E). Overall, capsaicin applied in the presence of chemerin also resulted in a significant increase in mEPSC frequency (P = 0.008, paired t-test, n = 10, Figure 1F). However, when the effect of chemerin on capsaicin potentiation of mEPSC frequency was assessed, it was found that while capsaicin significantly increased mEPSC frequency (P = 0.0001, 2-way repeated measures ANOVA, Figure 1G), this potentiation was significantly attenuated by chemerin (P = 0.031, 2-way repeated measures ANOVA, Bonferroni post-tests).

**Figure 2:**
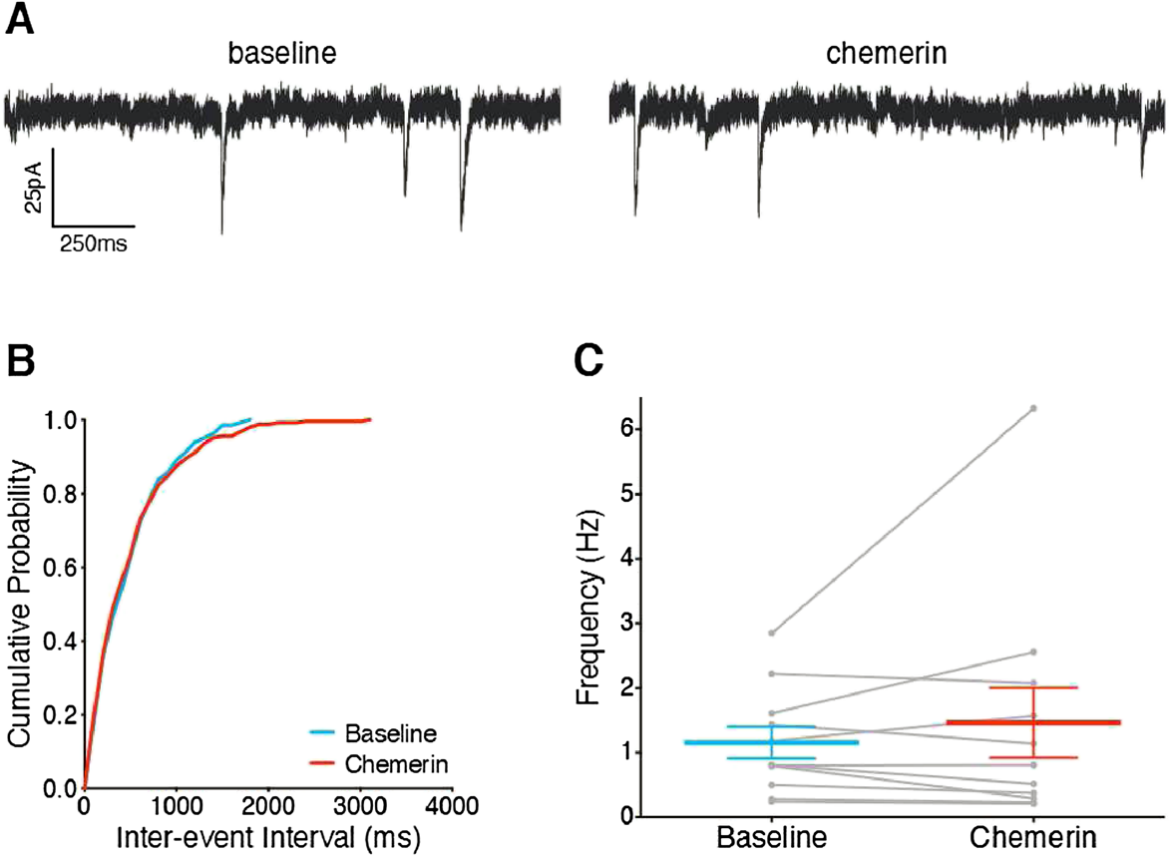
**Chemerin alone does not alter mEPSC frequency in lamina I NK1R+ neurons.** **A**. Representative mEPSC traces recorded prior to (‘baseline’, left) and during chemerin (100 ng/ml) application (right). **B**. Example cumulative probability curve shows chemerin does not alter the distribution of mEPSC inter-event intervals in an individual neuron (P = 0.992, 2-sample Kolmogorov-Smirnov test). **C**. Overall, chemerin does not alter mEPSC frequency in non-potentiated conditions (P = 0.824, Wilcoxon, n = 11). Data presented as mean ± SEM, grey points and lines in **C** indicate trajectories for individual neurons.

To assess whether chemerin altered mEPSC frequency in lamina I NK1R+ neurons in non-potentiated conditions, chemerin alone (100 ng/ml) was bath applied for 10 mins (example traces shown in Figure 2A). Chemerin alone resulted in no change in the distribution of mEPSC inter-event intervals in 6/11 neurons, while in 2/11 there was a significant leftward shift and in 3/11 a significant rightward shift (Kolmogorov-Smirnov 2-sample test, example shown in Figure 2B). Overall, chemerin alone did not alter mEPSC frequency (P = 0.824, Wilcoxon, Figure 2C) or amplitude (26.3 ±1.3 vs. 25.8 ±1.0 pA, P = 0.654, paired t-test, data not shown), indicating that chemerin does not alter basal excitatory input to lamina I NK1R+ neurons.

### Characterisation of monosynaptic C-fibre input to lamina I NK1R+ neurons

**Figure 3:**
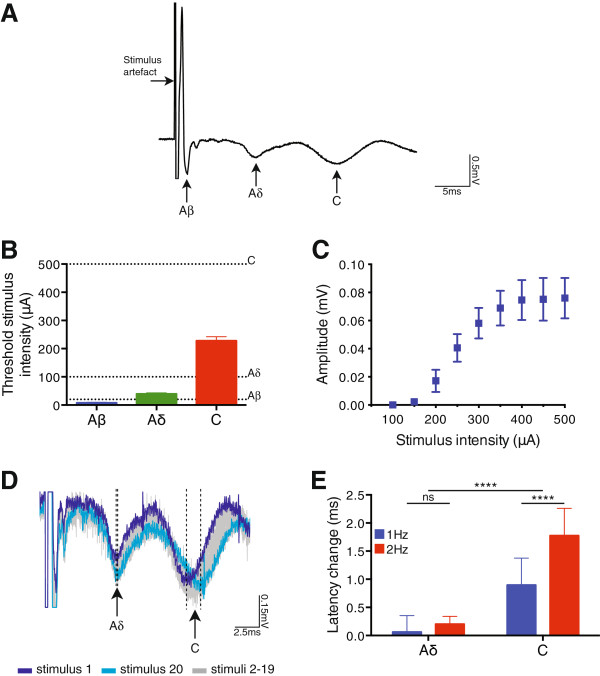
**Primary afferent fibre characterisation in compound action potential recordings.** **A**. Representative compound action potential trace recorded from an isolated dorsal root, evoked by 500 *μ*A stimulation, illustrating the fast (A *β*), medium (A *δ*) and slow (C) conducting components (average of 10 traces shown). **B**. Threshold stimulus intensity of the A *β*-, A *δ*- and C-fibre components, which differed significantly with fibre type (P < 0.0001, 1-way ANOVA, n = 9). **C**. Stimulus response relationship for the C-fibre component. **D**. 1 Hz stimulation of an isolated dorsal root resulted in a prolongation of latency in the C-, but not A *δ*-fibre component. Broken lines denote the negative peak of the A *δ*-/C-fibre response during the first and last stimuli. **E**. Quantification of the latency prolongation demonstrated that the C-fibre, but not A *δ*-fibre, component exhibited a significant frequency dependent latency prolongation (A *δvs*. C and 1 Hz *vs*. 2 Hz, P < 0.0001, 2-way ANOVA). All data presented as mean ± SEM. Dotted lines in **B**. indicate the dorsal root stimulation intensities (A *β*, 20 *μ*A; A *δ*, 100 *μ*A; C, 500 *μ*A) used in the subsequent patch-clamp electrophysiology studies. ∗∗∗∗ P < 0.0001.

To confirm the appropriate stimulation intensities to electrically activate, and thus enable the characterisation of, primary afferent input to lamina I NK1R+ neurons, extracellular compound action potential recordings were made from dorsal roots isolated from control rats. Notably, it has previously been established that CFA inflammation does not alter primary afferent threshold or conduction velocity in both adult [29,30] and juvenile rats [11]. Primary afferent components could be divided into three distinct groups, which corresponded to A *β*-, A *δ*- and C-fibres (Figure 3A). As expected [11,29,30], the threshold stimulus intensity differed between the primary afferent components (A *β* < A *δ* < C, P < 0.0001, 1-way ANOVA, n = 9, Figure 3B) as did the conduction velocity (A *β* 4.37 ± 0.48 m/s, A *δ* 0.83 ± 0.10 m/s, C 0.19 ± 0.01 m/s, P < 0.0001, 1-way ANOVA, n = 9). When the stimulus response relationship of the C-fibre component was assessed, it was found that the response amplitude gradually increased from an intensity of 150 *μ*A, indicating a gradual recruitment of C-fibres, and plateaued at an intensity of 400 *μ*A (Figure 3C), suggesting that the majority of C-fibres were activated at this intensity. It has been reported that in response to repetitive stimulation, the slow (C-fibre) component, but not A-fibre components, displays a frequency-dependent prolongation of latency [30]. In agreement with these findings, when isolated dorsal roots were repetitively stimulated at frequencies of 1 or 2 Hz, we observed a clear prolongation of latency in the C-fibre, but not A *δ*-fibre, component (representative traces in Figure 3D), with there being a significantly greater latency change in the C-fibre compared to the A *δ*-fibre component (P < 0.0001, 2-way ANOVA, Figure 3E). Furthermore, 2 Hz stimulation resulted in a significantly greater degree of latency prolongation than 1 Hz in the C-fibre component (P < 0.0001, 2-way ANOVA followed by Bonferroni post-tests, Figure 3E), indicating that this feature was frequency-dependent. Based on these data, 20, 100 and 500 *μ*A were used to activate the different primary afferent components in the subsequent patch-clamp recordings and are detailed in Figure 3B.

**Figure 4:**
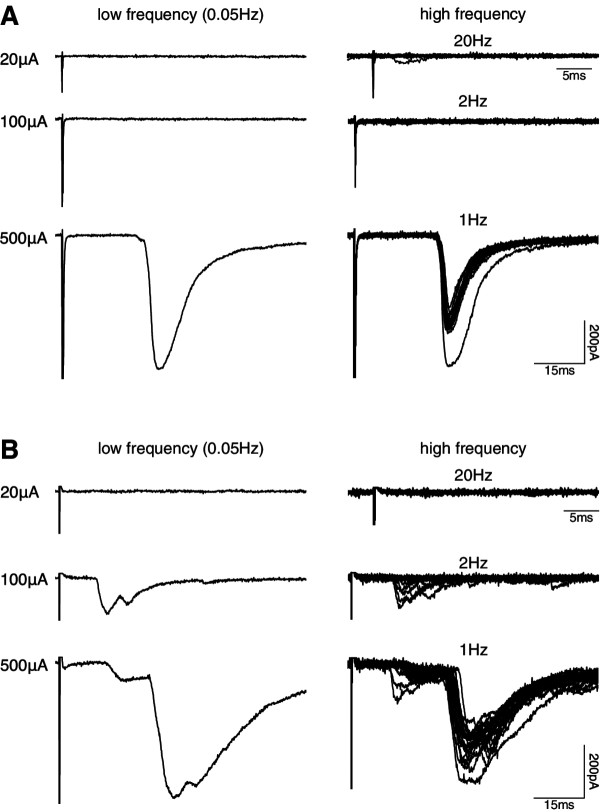
**Monosynaptic C-fibre input to lamina I NK1R+ neurons.** **A**. and **B**. Characterisation of primary afferent input to lamina I NK1R+ neurons receiving monosynaptic C-fibre only and monosynaptic C-fibre with polysynaptic A *δ*-fibre input, respectively. Left traces show examples of ESPCs evoked by dorsal root stimulation at A *β*- (20 *μ*A), A *δ*- (100 *μ*A) and C-fibre (500 *μ*A) intensities, at low frequency (0.05 Hz). Each trace is an average of 3 traces. Right traces show examples of EPSCs evoked using higher-frequency stimulation (20 *μ*A/20 Hz; 100 *μ*A/2 Hz; 500 *μ*A/1 Hz). Each trace comprises 20 superimposed traces.

Figure 4A shows an example of eEPSCs recorded from a lamina I NK1R+ neuron, in a CFA spinal cord slice, that received monosynaptic C-fibre input only. Stimulation of the dorsal root at low-frequency (0.05 Hz, duration 0.1 ms, three times, left traces) at A *β*-fibre (20 *μ*A) and A *δ*-fibre (100 *μ*A) intensities did not evoke EPSCs, whereas stimulation at C-fibre intensity (500 *μ*A) clearly evoked a response, which when stimulated at high-frequency (1 Hz, right traces) was reliably evoked, indicating that this C-fibre-evoked response was monosynaptic. An example of eEPSCs recorded from a lamina I NK1R+ neuron, in a control spinal slice, receiving monosynaptic C-fibre with polysynaptic A *δ*-fibre input is shown in Figure 4B. Low-frequency dorsal root stimulation at A *δ*-fibre, but not A *β*-fibre, intensity evoked small ESPCs, while C-fibre intensity additionally evoked a larger longer latency component. When stimulated at high-frequency (2 Hz), the A *δ*-fibre response displayed failures, indicating a polysynaptic input, while the C-fibre input was found to be monosynaptic as it was reliably evoked during 1 Hz stimulation.

### Chemerin attenuates monosynaptic C-fibre input to a subset of lamina I NK1R+ neurons in inflammatory pain

**Figure 5:**
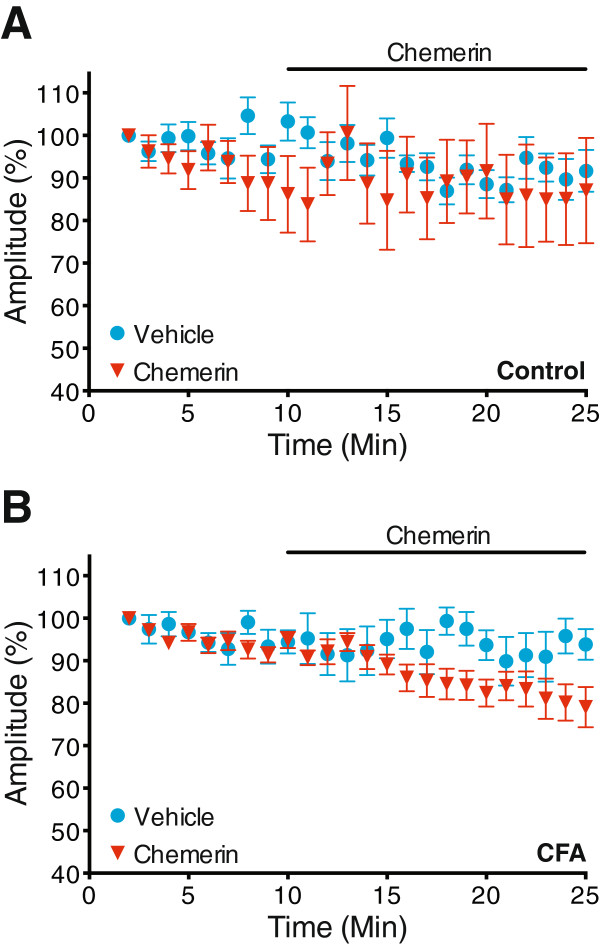
**Chemerin does not alter monosynaptic C-fibre eEPSCs in the overall lamina I NK1R+ neuronal population.** **A**. Chemerin (100 ng/ml) does not alter the peak amplitude of monosynaptic C-fibre eEPSCs in control tissue (P = 0.502, chemerin n = 7, vehicle n = 8), although there is a significant decline in peak amplitude over time (P = 0.013). **B**. In tissue isolated from CFA treated rats, there is no difference in the monosynaptic C-fibre eEPSCs between chemerin and vehicle groups (P = 0.152, chemerin n = 16, vehicle n = 7), although there is a significant reduction in peak amplitude over time (P < 0.0001) and an interaction between these factors (P = 0.0006). All statistics = 2-way repeated measures ANOVA. All data presented as mean ± SEM.

To determine whether chemerin modulates monosynaptic C-fibre input to lamina I NK1R+ neurons in inflammatory pain, C-fibre evoked excitatory postsynaptic currents (eEPSCs) were recorded in spinal cord slices from control (untreated) and CFA treated rats, prior to and during chemerin or vehicle application. In control neurons, a 15 minute application of chemerin (100 ng/ml) did not alter the peak amplitude of C-fibre eEPSCs when compared with vehicle (P = 0.502, 2-way repeated measures ANOVA, chemerin n = 7, vehicle n = 8, Figure 5A), although peak amplitude significantly declined over time (P = 0.013, 2-way repeated measures ANOVA). Likewise, no significant difference was detected in the peak amplitude of monosynaptic C-fibre eEPSCs between chemerin and vehicle application in CFA neurons (P = 0.152, 2-way repeated measures ANOVA, chemerin n = 16, vehicle n = 7, Figure 5B), but there was a significant reduction in peak amplitude over time (P < 0.0001, 2-way repeated measures ANOVA). However, there was a significant interaction between these factors (P = 0.0006, 2-way repeated measures ANOVA), indicating that the decline in peak amplitude was influenced by chemerin, which could be indicative of chemerin affecting a subset of neurons in inflammatory pain.

**Figure 6:**
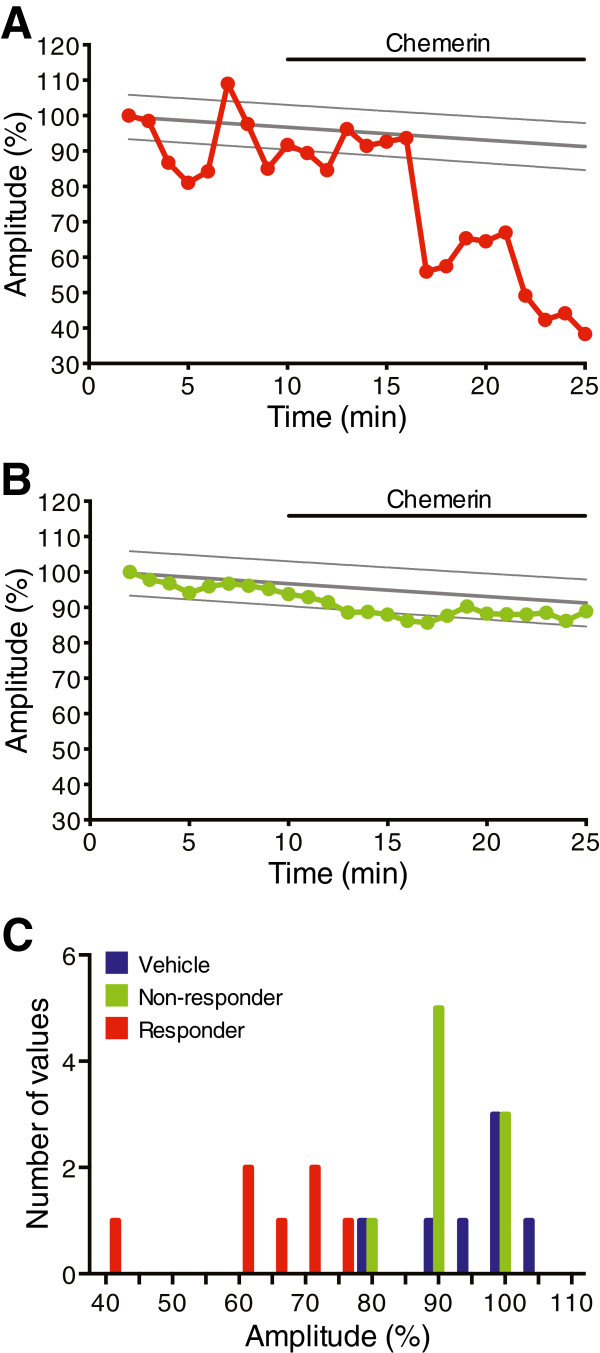
**Identification of a subpopulation of chemerin-responsive lamina I NK1R+ neurons.** **A**. Example of a neuron classified as a chemerin responder, because the peak amplitude of the C-fibre eEPSCs falls below the lower 95% prediction band (95% prediction bands designated by the upper and lower grey lines) for at least the final 5 mins of chemerin (100 ng/ml) application. **B**. Example of a lamina I NK1R+ neuron classified as a chemerin non-responder because the peak amplitude of the C-fibre eEPSCs largely falls within the 95% prediction bands. **C**. Frequency histogram of the normalised mean eEPSC peak amplitude during the final 5 mins, demonstrating that the distribution of the responses of chemerin responders does not overlap with either the non-responder or vehicle groups.

Although chemerin did not alter C-fibre eEPSCs compared to vehicle when the entire population of CFA neurons was considered, because ChemR23 is only expressed on a subset of TRPV1+ and a subset of substance P+ afferents [14], which is likely to include C-fibre input to lamina I NK1R+ neurons [18-21], we hypothesised that chemerin may only be acting upon monosynaptic C-fibre input to a subset of these neurons. To identify this subset, linear regression analysis was performed on vehicle data to calculate 95% prediction bands. Neurons were classified as responders if the eEPSC peak amplitude fell below the lower 95% prediction band for at least the final 5 mins of chemerin treatment (example in Figure 6A). Neurons where this did not occur were classified as non-responders (example in Figure 6B). Using this criteria it was revealed that in CFA inflammation, 7/16 neurons were classified as responders, while 9/16 were non-responders. Notably, when this criteria was applied to control neurons, only 1/7 was classified as a responder, while the remaining neurons were non-responders. To justify this classification and confirm that responders and non-responders were distinct populations, frequency histograms of normalised mean eEPSC peak amplitude in the final 5 mins of chemerin/vehicle application, recorded from CFA tissue were plotted (Figure 6C). Importantly, this clearly shows that the distribution of the responses of chemerin responders did not overlap with either the non-responder or vehicle groupings, validating the use of this classification.

**Figure 7:**
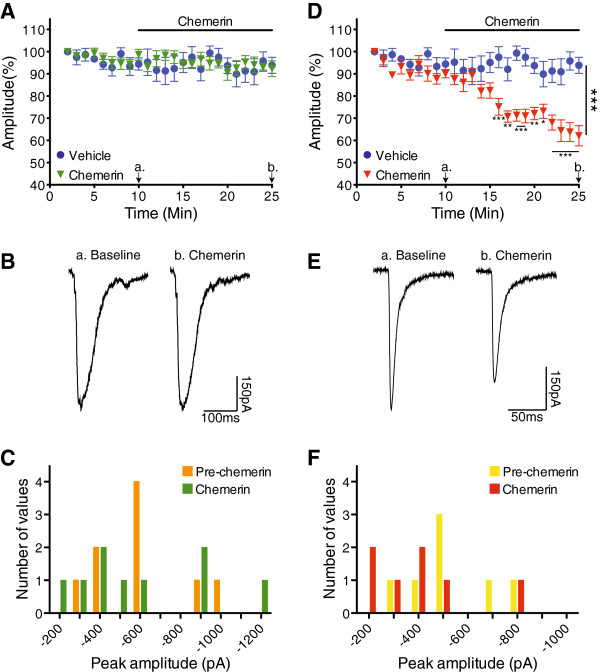
**Chemerin attenuates C-fibre input to a subset of lamina I NK1R+ neurons in CFA inflammation.** **A**. Chemerin has no effect on the peak amplitude of monosynaptic C-fibre eEPSCs in the population of neurons classified as ‘non-responders’ (P = 0.802, 2-way repeated measures ANOVA, representative traces shown in **B**). In this subgroup of non-responders, chemerin did not alter the distribution of actual C-fibre eEPSC peak amplitudes (P = 0.983, paired t-test, **C**). **D**. In the subgroup of chemerin responders, chemerin significantly reduces the C-fibre eEPSC amplitude (P = 0.001, 2-way repeated measures ANOVA, representative traces shown in **E**). Furthermore, in neurons classified as chemerin responders, chemerin resulted in a significant leftward shift in the distribution of actual C-fibre eEPSC peak amplitudes (P = 0.028, paired t-test, **F**). Example traces show an average of 3 sweeps, baseline and chemerin traces recorded at points ‘**a.**’ and ‘**b.**’ respectively, denoted by arrows on relevant graphs. Vehicle n = 7, chemerin responders n = 7, chemerin non-responders n = 9. Data displayed as mean ± SEM. ∗ P < 0.05, ∗∗ P < 0.01, ∗∗∗ P < 0.001.

The C-fibre eEPSC data recorded in CFA tissue were accordingly separated into ‘responders’ and ‘non-responders’ and compared to the vehicle group. In the subgroup of neurons classified as non-responders, chemerin did not alter the peak amplitude of C-fibre eEPSCs (P = 0.802, 2-way repeated measures ANOVA, Figure 7A, example traces in Figure 7B). In those neurons classified as responders, application of chemerin significantly attenuated the peak amplitude of the monosynaptic C-fibre eEPSCs in these neurons (P = 0.001, 2-way repeated measures ANOVA, Figure 7D, example traces in Figure 7E). Post-tests specifically revealed that chemerin significantly inhibited the peak amplitude of the monosynaptic C-fibre eEPSCs during the final 10 mins of chemerin application (P < 0.05 to P < 0.001, 2-way repeated measures ANOVA, Bonferroni post-tests). Importantly, the input resistance of both responder and non-responder groups was stable throughout the recording period (no significant change with respect to time, P = 0.392, 2-way ANOVA). When the distribution of the actual mean peak amplitude of monosynaptic C-fibre eEPSCs, recorded in the 5 mins prior to and the final 5 mins of chemerin application, was compared, it was similarly found that chemerin significantly altered the eEPSC peak amplitude distribution in responders (P = 0.028, paired t-test, Figure 7F), but not non-responders (P = 0.983, paired t-test, Figure 7C).

#### Chemerin presynaptically inhibits monosynaptic C-fibre input to a subset of lamina I NK1R+ neurons in inflammatory pain

**Figure 8:**
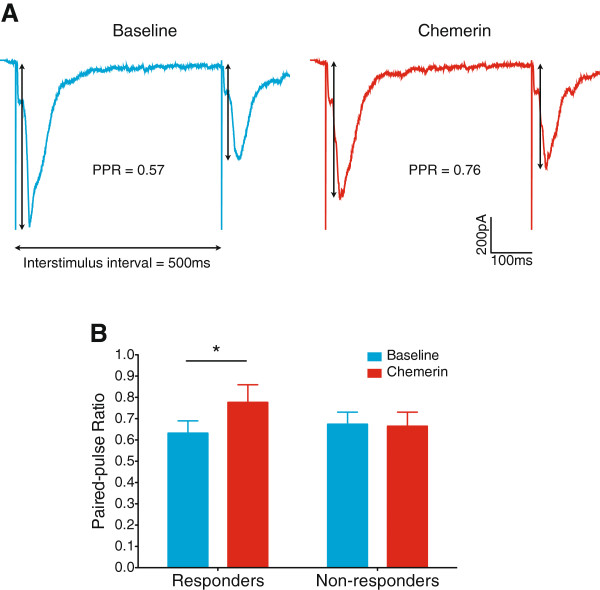
**Chemerin reduces paired-pulse depression of monosynaptic C-fibre input to lamina I NK1R+ neurons.** **A**. Representative EPSC traces evoked by C-fibre paired-pulse stimulation in a chemerin responder recorded before (left, blue trace) and during (right, red trace) chemerin application (average of 6 sweeps shown), where chemerin has increased the paired-pulse ratio/decreased paired-pulse depression. **B**. In neurons classified as chemerin responders, application of chemerin significantly increases the paired-pulse ratio/decreases paired-pulse depression (P = 0.031, 2-way repeated measures ANOVA, followed by Bonferroni post-tests). Responders n = 5, non-responders n = 6. Data presented as mean ± SEM. ∗ P < 0.05.

Previous data have suggested that the ChemR23 agonist, RvE1, inhibits potentiated spinal cord responses via both pre and postsynaptic mechanisms [14]. Therefore, to determine the site of action of the chemerin inhibition of monosynaptic C-fibre input to lamina I NK1R+ neurons seen here, paired-pulse recordings were conducted in a subset of neurons from CFA treated rats prior to (‘baseline’) and during chemerin application. Paired-pulse stimulation of monosynaptic C-fibre input to lamina I NK1R+ neurons resulted in paired-pulse depression (PPD) (example traces in Figure 8A). In neurons classified as responders, chemerin significantly increased the paired-pulse ratio (PPR) (P = 0.031, 2-way repeated measures ANOVA, Bonferroni post-tests, Figure 8B), indicating a significant decrease in PPD. In non-responders, chemerin did not alter the PPR (P >0.999, 2-way repeated measures ANOVA, Bonferroni post-tests). These findings strongly suggest that chemerin acts presynaptically to inhibit monosynaptic C-fibre input to a subset of lamina I NK1R+ neurons. Furthermore, the example traces in Figure 8A were obtained from a lamina I NK1R+ neuron which received both monosynaptic C-fibre and polysynaptic A *β*-fibre input. However, chemerin did not alter the amplitude of this polysynaptic input (data not shown), further supporting a presynaptic site of chemerin action, given that a postsynaptic effect would likely influence the response to both types of input.

## Discussion

It is known that ChemR23 agonists can alleviate hypersensitivity in animal models of inflammatory pain through both peripheral and central mechanisms [14]. These include a reduction in peripheral inflammation and a normalisation of potentiated spinal cord responses, respectively. In terms of central mechanisms, electrophysiological recordings from unidentified lamina II dorsal horn neurons have demonstrated that the ChemR23 agonists, RvE1 and chemerin, attenuate capsaicin potentiation of sEPSC frequency. However, as these findings were obtained using sEPSC recordings in unidentified neurons it is not known where in the spinal cord network or upon which neuronal subtypes that these effects are mediated. In this study we have investigated the ability of chemerin to modulate excitatory input to lamina I NK1R+ neurons following capsaicin potentiation and CFA inflammation. Our results have novelly revealed that chemerin can attenuate capsaicin potentiation of mEPSC frequency in lamina I NK1R+ neurons and presynaptically reduce monosynaptic C-fibre input to a subset of these neurons in inflammatory pain. Notably, chemerin was without effect in non-potentiated/control conditions. Given the essential role of lamina I NK1R+ neurons in the manifestation of inflammatory pain [7], which is driven by C-fibres [8], the chemerin attenuation of monosynaptic C-fibre input to these neurons suggests that the reported ability of ChemR23 agonists to attenuate inflammatory pain hypersensitivity may in part be due to presynaptic inhibition of monosynaptic C-fibre input to these key spinal cord output neurons.

We have demonstrated that chemerin can significantly reduce, but not eliminate, capsaicin potentiation of mEPSC frequency in rat lamina I NK1R+ neurons. However, Xu et al. [14] report that chemerin, at the same dose used here, can completely prevent capsaicin potentiation of sEPSC frequency in unidentified mouse lamina II neurons. This difference likely reflects the different concentrations of capsaicin employed, 1 *μ*M in the present study, which resulted in a ∼44-fold increase in frequency, vs. 100 nM used by Xu et al. [14], which increased frequency by only 2-fold. We did investigate use of 100 nM capsaicin, but found that this concentration did not reliably potentiate mEPSC frequency in lamina I NK1R+ neurons (data not shown). This dissimilarity may also reflect the different species employed, rat vs. mouse, different cell types targeted, lamina I NK1R+ neurons vs. unidentified lamina II neurons or the different recording approach, mEPSC vs. sEPSC recording, employed. Other groups have demonstrated that 100 nM capsaicin can potentiate mEPSC frequency in unidentified lamina I/II neurons [31,32], however potentiation of mEPSCs in lamina I NK1R+ neurons has only been reported using 1 *μ*M capsaicin [19]. Interestingly, Labrakakis and MacDermott [19] report that 73% of lamina I NK1R+ neurons display an increase in mEPSC frequency in response to 1 *μ*M capsaicin, which is comparable to our findings, that 83% of neurons had capsaicin-sensitive input.

On the basis of anatomical expression data [14] we hypothesised that ChemR23 would be expressed on a subset of monosynaptic C-fibre inputs to lamina I NK1R+ neurons. Indeed we provide supporting functional evidence for this expression pattern through our novel demonstration that chemerin presynaptically attenuates monosynaptic C-fibre input to a subset of these neurons in inflammatory pain. To unequivocally demonstrate that the chemerin effects reported here are mediated via ChemR23, we would ideally have shown blockade by a ChemR23 antagonist, but this was not possible as no such ligand is commercially available. Pertussis toxin (PTX), an inhibitor of G_*α*_i coupled GPCRs, the receptor family to which ChemR23 belongs, has been used by others to inhibit the RvE1 attenuation of capsaicin potentiated input in unidentified lamina II neurons [14]. While this approach could have been used to provide additional confirmation that the actions of chemerin were mediated by ChemR23, PTX inhibition of the chemerin response would only indicate that chemerin acted via a G_*α*_i coupled GPCR and not ChemR23 specifically.

ChemR23 agonists are proposed to reduce inflammatory pain in part by normalising potentiated spinal cord responses [14,33]. In the present data, chemerin reduced monosynaptic C-fibre input to a subset (∼44%) of lamina I NK1R+ neurons in CFA tissue but was without effect in control tissue. Interestingly, electrical stimulation of monosynaptic C-fibre input to lamina I projection neurons, that are likely to be NK1R+ neurons, in a manner which mimics the spontaneous firing pattern seen during inflammatory pain, results in the potentiation of C-fibre input to only a subset of these neurons [12]. It could therefore be hypothesised that neurons classified as chemerin responders were the subset that received potentiated C-fibre input. We therefore investigated whether there was a correlation between the initial peak amplitude of monosynaptic C-fibre eEPSCs (with greater amplitudes possibly signifying potentiated inputs) and the magnitude of the chemerin mediated change in peak amplitude. Our results found there to be no correlation between the initial eEPSC peak amplitude and the amplitude change (data not shown), however it should be recognised that this finding cannot confirm or refute the possibility that neurons classified as chemerin responders were those that received potentiated input, as it is not possible to directly distinguish potentiated inputs in this kind of population comparison study. Moreover, when the peak amplitude of monosynaptic C-fibre eEPSCs are compared between control and CFA tissue there is no significant potentiation observed overall (data not shown) as previously reported [11].

It has previously been established that ChemR23 agonists do not alter acute pain sensitivity and have no effect upon sEPSC frequency, in unidentified lamina II neurons, in non-potentiated conditions [14]. In accordance, we have shown that chemerin does not alter basal mEPSC frequency or amplitude in lamina I NK1R+ neurons or the peak amplitude of monosynaptic C-fibre eEPCSs in these neurons in control tissue. It is proposed that ChemR23 activation reduces inflammatory pain hypersensitivity by normalising potentiated spinal cord responses, as opposed to a general reduction in sensory transmission [14,33], for which our findings provide additional support.

ChemR23 activation is proposed to normalise spinal cord inflammatory pain potentiation by inhibition of the extracellular signal-regulated kinase (ERK) pathway, which is key for central sensitisation [34], both presynaptically in the central terminals of primary afferents and postsynaptically in dorsal horn neurons [14]. Inhibition of ERK, with the mitogen-activated protein kinase kinase inhibitors PD98059 and U0126, prevents capsaicin potentiation of sEPSCs, while application of RvE1 reduces capsaicin/TNF- *α* driven ERK phosphorylation in DRG cultures [14]. Therefore, the chemerin attenuation of capsaicin potentiated mEPSC frequency and chemerin mediated presynaptic inhibition of monosynaptic C-fibre inputs in inflammatory pain, reported here, may result from blockade of ERK mediated glutamate release from central terminals. Chemerin may also mediate its effects via inhibition of TRPV1, which is crucial for inflammatory pain [35,36], as RvE1 is a highly potent inhibitor of TRPV1, with different resolvins interestingly differentially modulating different TRP channels via GPCR activation [37,38].

Chemerin is a natural ChemR23 ligand [15,16] and while it is currently unclear which cell types are responsible for its endogenous production and release, endothelial cells, keratinocytes, chondrocytes, platelets and osteoclasts have all been proposed as possible sources [39-44]. Chemerin plays a key role in a number of physiological processes including adipocyte generation and metabolism [45] and the chemotaxis of macrophages and dendritic cells [16]. However, it is not currently known whether endogenous chemerin plays any role in the modulation of inflammatory pain.

It is worth noting that chemerin is known to display high affinity binding with receptors other than ChemR23, namely the chemokine (C-C motif) receptor-like 2 (CCRL2) and G protein-coupled receptor 1 (GPR1), however these receptors are thought to play a limited role in cell signalling. Current evidence suggests that CCRL2 is not involved in cell signalling but may play a functional role in presenting chemerin to ChemR23 [40,46,47]. Interestingly, CCRL2 is known to be expressed in the spinal cord and wider CNS, predominantly in microglia [48]. Binding of chemerin to GPR1 results in receptor internalisation and signalling, but this signalling is weak [49] and it has been speculated that GPR1 may act as a decoy receptor [40]. There is evidence that GPR1 is expressed in the CNS [50-53], however spinal cord expression has not been investigated. It is possible, therefore, that inflammation-induced changes in the expression of either of these receptors could have influenced the results presented here.

While the evidence presented here supports the work by Xu et al. [14] which revealed that drugs which target ChemR23 may be effective in the treatment of inflammatory pain, it is worth noting that chemerin or RvE1 may have limited therapeutic potential given that they are not metabolically stable and are rapidly inactivated in vivo [33,54,55]. Interestingly, a stable analogue of RvE1, 19-(p-fluorophenoxy)-RvE1, has been shown to effectively reduce thermal hypersensitivity in the CFA inflammatory pain model for an extended time period compared to RvE1 [14]. Stable chemerin analogues have been developed [56], however their use has not yet been investigated in models of inflammatory pain. Further research into the use of these or new stable analogues of ChemR23 agonists should further establish ChemR23 as a promising target for the treatment of inflammatory pain.

## Conclusions

This study demonstrates that the ChemR23 agonist, chemerin, can attenuate capsaicin potentiation of mEPSC frequency in lamina I NK1R+ neurons, but is without effect in non-potentiated conditions. Furthermore, chemerin presynaptically inhibits monosynaptic C-fibre input to a subset of these neurons in inflammatory pain, but does not alter C-fibre eEPSCs in control conditions. These findings suggest that the reported ability of ChemR23 agonists to attenuate inflammatory pain hypersensitivity could in part be due to the inhibition of C-fibre input to these key spinal cord output neurons and provides further evidence that ChemR23 represents a promising target for the development of novel inflammatory pain treatments.

## Methods

### Animals

All procedures were approved by the University of Edinburgh Ethical Review Committee and carried out in accordance with the UK Animals (Scientific Procedures) Act 1986. Juvenile Sprague Dawley rats of both sexes (approximately postnatal day 21 [P21]; the University of Edinburgh Biological Research Resources, Edinburgh UK) were used in all experiments. No differences were found between male and female rats so all data presented are a combination of both sexes. Animals were housed in cages at 21°C and 55% relative humidity, with a 12 h light-dark cycle. Food and water were provided ad libitum.

### Inflammatory pain model

Complete Freund’s adjuvant (CFA, 0.5 mg/ml saline) was injected into the intraplantar surface of the left hindpaw (1 *μ*l/g body weight) under isoflurane anaesthesia at ∼P18, 2–6 days prior to electrophysiological recordings at ∼P21. This procedure has been shown to produce persistent peripheral inflammation and behavioural hypersensitivity in juvenile rats [11].

### Spinal cord slice preparation

CFA treated or naïve untreated (control) rats were anaesthetised with isoflurane and decapitated. Spinal cords, in some cases with dorsal roots attached, were removed and were initially placed in ice-cold dissection solution. The lumbar (L4/L5) segment was embedded in an agarose block and cut into transverse slices (350 *μ*m). Where slices with dorsal roots attached were cut, dorsal root ganglia were removed and only the left dorsal roots were used in CFA treated rats. Slices were incubated at 36–37°C for 1 h in oxygenated recovery solution after which they were incubated at room temperature for 30 mins with 35 nM tetramethylrhodamine conjugated substance P (TMR-SP), as described previously [10,11,19]. Slices were allowed to recover for a further 1 h at room temperature prior to recording. Slices were transferred to the recording chamber of an upright microscope (Zeiss), equipped with fluorescence for the identification of TMR-SP labelled (TMR-SP+) neurons and infrared-differential interference contrast (IR-DIC) for electrophysiological recordings and were continually perfused with oxygenated Krebs solution (1–2 ml/min) at room temperature. The composition of the 95% O_2_/5% CO_2_ saturated Krebs solution is as follows (in mM): 125 NaCl, 2.5 KCl, 26 NaHCO_3_, 1.25 NaH_2_PO_4_, 25 glucose, 1 MgCl_2_, 2 CaCl_2_, pH7.4. Recovery solution was identical to Krebs but with 6 mM MgCl_2_ and 1.5 mM CaCl_2_. Dissection solution was the same as recovery but with the addition of 1 mM kynurenic acid.

Our technique for pre-identifying NK1R+ neurons is expected to have little impact on the synaptic activity we are studying. TMR-SP is one of the least biologically active of fluorescence conjugated substance P analogues and does not inhibit neuronal M-type K^+^ current at the nanomolar concentration used in our study, although it does elevate calcium in Chinese hamster ovary cells expressing NK1 receptor [57]. Moreover, this TMR-SP labelling approach has been validated in a previous study of spinal NK1R+ neurons [20]. However, we cannot rule out the possibility that this labelling approach may interfere with the response of these neurons to chemerin. Retrograde labelling of these likely projection neurons [12,58] is an alternative approach that could be employed to address this potential issue.

### Patch-clamp recording

Whole-cell patch-clamp recordings were made from ‘identified’ neurons in the lamina I region of the dorsal horn. All recordings were made at a holding potential of -70 mV and junction potential was corrected prior to recording. Data were recorded and acquired with an Axopatch 200B amplifier and pClamp 10 software (Molecular Devices). Data were filtered at 5 kHz and digitised at 10 kHz.

#### mEPSC recording

mEPSCs were recorded from lamina I NK1R+ neurons in spinal slices from control tissue with dorsal roots removed, in the presence of 0.5 *μ*M TTX, 10 *μ*M bicuculline and 1 *μ*M strychnine. The intracellular solution composition was as follows (in mM); 110 K-methanesulfonate, 10 NaCl, 10 EGTA, 1 CaCl_2_, 10 HEPES, 5 Mg^2+^-ATP, 0.5 Na^+^-GTP, pH adjusted to 7.2 with KOH, osmolarity 290 mOsm. 1 *μ*M Alexa Fluor 488 hydrazide was also included in the recording pipette to confirm that the TMR-SP+ neuron detected under fluorescence was the same as that targeted for recording under IR-DIC.

Baseline mEPSCs were recorded for 5 mins, following which the TRPV1 agonist, capsaicin (1 *μ*M), was bath applied for 5 mins to pharmacologically potentiate excitatory input [59]. Potentiation was assessed by comparing mEPSCs recorded at baseline (final 2 mins) and during capsaicin application (final 2 mins). To assess whether chemerin modulates this capsaicin potentiation, chemerin (100 ng/ml) was bath applied for 10 mins prior to and throughout capsaicin application, in a separate group of lamina I NK1R+ neurons. Two separate neuronal populations were used, rather than a within cell comparison, because capsaicin potentiation of mEPSC frequency does not return to baseline, even following a long wash period (data not shown), as is reported elsewhere [31] and it is known that repeated capsaicin applications results in a progressive reduction in capsaicin response [31,60]. The effect of chemerin upon mEPSCs in non-potentiated conditions was similarly assessed by applying chemerin alone. In all cases the final 2 mins of baseline/drug application was analysed using Mini Analysis (Synaptosoft), mEPSC events were automatically detected by the software and were then accepted or rejected following further visual examination.

#### eEPSC recording

eEPSCs were recorded from lamina I NK1R+ neurons in spinal cord slices, with dorsal roots attached, from control and CFA treated rats. Monosynaptic C-fibre input was identified by stimulation of the dorsal root with a suction electrode, as described previously [10,11]. The dorsal root was stimulated (3 times) at low frequency (0.05 Hz), using an ISO-Flex stimulus isolator (A.M.P.I Intracel), at 20, 100 and 500 *μ*A (0.1 ms stimulus duration) to activate A *β*-, A *δ*- and C-fibre inputs, respectively. C-fibre eEPSCs were identified as longer latency components evident at 500 *μ*A (but not 20 or 100 *μ*A) stimulation intensity. C-fibre eEPSCs were considered monosynaptic if when stimulated (20 times) at the higher frequency of 1 Hz they displayed no synaptic failures, regardless of whether there was latency variability [30]. A 500 *μ*A stimulation intensity was employed for C-fibres, as compound action potential recordings demonstrated that the slow C-fibre component appeared to be maximally recruited at this intensity (Figure 3C). It is possible that higher stimulation intensities could recruit additional C-fibres, but this seems unlikely given that this 500 *μ*A intensity reveals monosynaptic C-fibre input to a similar proportion of these neurons [11] as that observed in experiments employing stimulation intensities greater than 800 *μ*A [9]. The intracellular solution was composed of the following (in mM): 120 Cs-methylsulfonate, 10 Na-methylsulfonate, 10 EGTA, 1 CaCl_2_, 10 HEPES, 5 QX-314-Cl [2(triethylamino)-N-(2,6-dimethylphenyl) acetamine chloride] and 2 Mg^2+^-ATP, pH adjusted to 7.2 with CsOH, osmolarity 290 mOsm. Additionally, 1 *μ*M Alexa Fluor 488 hydrazide was included in the recording pipette.

To assess the ability of chemerin to modulate monosynaptic C-fibre input to lamina I NK1R+ neurons, C-fibre EPSCs were evoked at 500 *μ*A (0.05 Hz, 0.1 ms stimulus duration) for 10 mins (‘baseline’), followed by a further 15 mins in the presence of either chemerin (100 ng/ml) or vehicle (Krebs only). Peak monosynaptic C-fibre eEPSC amplitude was measured for each sweep and the mean peak amplitude per minute was calculated. All data were normalised to minute 2 because in many neurons there was a large degree of run-down in the eEPSC peak amplitude between minute 1 and minute 2, after which the response generally stabilised (data not shown).

We hypothesised that chemerin would modulate primary afferent input to a subset of lamina I NK1R+ neurons, given that ChemR23 is only expressed on a subset of TRPV1+ and a subset of substance P+ afferents [14]. To identify this subgroup, linear regression analysis was performed on vehicle data to calculate 95% prediction bands. A neuron was classified as a responder if the eEPSC peak amplitude fell below the lower limit of the 95% prediction bands for at least the final 5 mins of chemerin application. To evaluate the validity of this classification method and to assess whether responders and non-responders were two distinct subpopulations, frequency histograms of normalised mean peak amplitude in the final 5 mins of chemerin/vehicle application were plotted. Additionally, histograms of actual mean peak amplitude in the 5 mins prior to and the last 5 mins of chemerin application were plotted for both chemerin responders and non-responders.

To determine the pre/postsynaptic nature of chemerin effects, paired-pulse recordings were conducted in a subset of neurons in CFA tissue. Monosynaptic C-fibre eEPSCs were recorded at baseline and in the presence of chemerin as described above, however rather than a single stimulus being applied every 20 seconds (0.05 Hz) the dorsal root was stimulated twice in close succession (500 ms interstimulus duration) every 20 seconds. PPR, of the monosynaptic C-fibre component, was calculated for the 5 mins prior to (‘baseline’) and the final 5 mins of chemerin application, as PPR = mean eEPSC peak amplitude 2/mean eEPSC peak amplitude 1, so as to correct for misleading facilitation that can be caused by random amplitude fluctuations [61].

### Isolated dorsal root preparation

Spinal cords were removed from control rats, in the manner described above and lumbar (L4/L5) dorsal roots, with dorsal root ganglia removed, were cut near to the dorsal root entry zone and placed in oxygenated recovery solution, at a temperature of 36–37°C, for 1 h and were then maintained at room temperature prior to recording.

### Compound action potential recording

Compound action potential recordings were made from isolated dorsal roots using two glass suction electrodes, placed at either end of the dorsal root. To determine the electrical activation threshold of the different primary afferent components, dorsal roots were stimulated 10 times at a frequency of 0.2 Hz (0.1 ms duration) with an ISO-flex stimulus isolator (A.M.P.I. Intracel) at the following intensities (in *μ*A): 1, 2, 3, 4, 5, 7.5, 10, 15, 20, 25, 30–100 (in 10 *μ*A steps) and 150–500 *μ*A (in 50 *μ*A steps) [11]. The main components of the compound action potentials were differentiated as fast (A *β*), medium (A *δ*) and slow (C) conducting components, each with a characteristic triphasic (positive-negative-positive) response (Figure 3A). Threshold stimulus intensities for the components were defined as the lowest stimulation intensity at which the negative component of the triphasic response was first clearly identifiable. The conduction velocity of each component was calculated based on the latency to the negative peak of the response, at 20, 100 and 500 *μ*A for A *β*-, A *δ*- and C-fibres, respectively. The amplitude of the C-fibre component was measured as the distance between the negative and positive peaks, at 500 *μ*A. To assess latency prolongation in the A *δ*- and C-fibre components, dorsal roots were stimulated 20 times at 1 and 2 Hz (500 *μ*A intensity, 0.1 ms duration) and the difference between the latency measured at stimulus 1 and stimulus 20 was calculated. Data were acquired and recorded using a Cygnus ER-1 differential amplifier (Cygnus Technologies Inc.) and pClamp 10 software (Molecular Devices).

### Statistical analysis

All data were assessed for normality using D’Agostino and Pearson omnibus normality tests, to determine whether it was appropriate to use parametric or non-parametric statistical tests and are presented as mean ± SEM. All statistical analysis was performed using Prism 6 (Graphpad Software).

### Materials

All chemicals were obtained from Sigma except; bicuculline (Tocris), TMR-SP (Enzo Life Sciences), Alexa Fluor 488 hydrazide (Molecular Probes), chemerin (R&D Systems), QX-314-Cl and TTX (Alomone Labs).

## Competing interests

The authors declare that they have no competing interests.

## Authors’ contributions

Data collection and analysis was carried out by ACD. All authors contributed to the conception and design of the study, interpretation of the data and the writing and editing of the manuscript. Both authors read and approved the final manuscript.
